# Eyesight of School Children

**Published:** 1883-07

**Authors:** 


### Eyesight of School Children.

The *Brit. Med. Jour.*, April 28, 1883, says that a lecture on the effect of reading and writing on the eyesight of young children was recently given at Berne by Professor Pfluger. The lecturer first called attention to the portentous fact that more than one half of 45,000 children lately examined in Germany were found to be suffering from defective vision. In some schools, the proportion of the short-sighted was as high as seventy to eighty per cent., whilst, in the Heidelberg Gymnasium, every lad in the school had bad eyesight. This lamentable state of things arose from an insufficiently lighted school-room, bad print, and bad paper, the method of writing in vogue, and ill-contrived desks and forms. The burdening of children with too many lessons, and the consequent restriction of their hours of play, is a still more potent cause of defective vision. In order to solve the vexed question of the influence of German caligraphy on the eyes of those who adopt it, the Government of Wurtemburg, some time ago appointed a commission, consisting of three schoolmasters and three physicians, to investigate the matter, and make a report. In the opinion of these gentlemen, the mere writing is least among the causes which unfavorably affect children's eye-sight. They found that, while comparatively few children write with their backs bent towards the left, fully eighty per cent give their back, when writing, a right inclination. The latter position tends to produce a permanent elevation of the right shoulder, and ultimately causes curvature of the spine. In the schools they visited, the commissioners actually found twenty per cent of the boys, and from thirty to forty per cent of the girls suffering from more or less pronounced curvatures due to this cause. The difference between the two sexes is probably due to the fact that lads, besides being more energetic in play, are more rationally clad than girl scholars. As to position in writing, the distance between the desk and the eyes ought to be about 25 centimetres (nearly 10 inches); yet it was rarely, indeed, that the commissioners met with any children who could keep their eyes at this distance from the paper. Many of them found it necessary to bring their faces within seven centimetres (2.75 inches) of their copy-books. The general conclusion of the commissioners, as of Professor Pfluger, is that of all the evils enumerated, the worst, and those most in need of reform, are the seats and desks at present in use. The Professor further remarked that only ten per cent of the children examined were naturally short-sighted, and that, as among wild races defective vision is almost entirely unknown, the trouble in question is peculiar to modern civilization and the existing system of teaching. Professor Pfluger expressed the fear that he was like one crying in the wilderness, the prevailing tendency being to lay on the children of this generation still heavier burdens, and to force their minds to the lasting injury of their bodies.

---

The essential oil of mustard is an excellent remedy for toothache arising from caries. Take a bit of cotton, moisten it with the oil, and insert it into the cavity. Relief is almost instantaneous.

---

It is said that Charles Reed, the novelist, named his dog Tonic, because it was a mixture of bark, steal, and whine.

